# (*E*)-2-(4-Benz­yloxy-2-hy­droxy­benzyl­idene)-*N*-phenyl­hydrazinecarbothio­amide

**DOI:** 10.1107/S1600536811049658

**Published:** 2011-11-25

**Authors:** K. Nisha, M. Sithambaresan, M. R. Prathapachandra Kurup

**Affiliations:** a Department of Applied Chemistry, Cochin University of Science and Technology, Kochi 682 022, India; b Department of Chemistry, Faculty of Science, Eastern University, Sri Lanka, Chenkalady, Sri Lanka

## Abstract

The title compound, C_21_H_19_N_3_O_2_S, exists in the thione form. The configuration about the C=N bond is *E*. The hydrazinecarbothio­amide unit adopts an almost planar arrangement, with maximum deviations of 0.016 (3) and −0.016 (2) Å for the two thio­urea N atoms. An intra­molecular O—H⋯N hydrogen bond occurs. Weak inter­molecular N—H⋯S, C—H⋯O and C—H⋯π inter­actions are observed in the crystal structure.

## Related literature

For applications of hydrazinecarbothio­amide and its derivatives, see: Casas *et al.* (2000 ▶); Lukevics *et al.* (1995 ▶). For the synthesis, see: Joseph *et al.* (2004 ▶). For related hydrazine­carbothio­amide structures, see: Seena *et al.* (2008 ▶). For standard bond lengths, see: Allen *et al.* (1987 ▶).
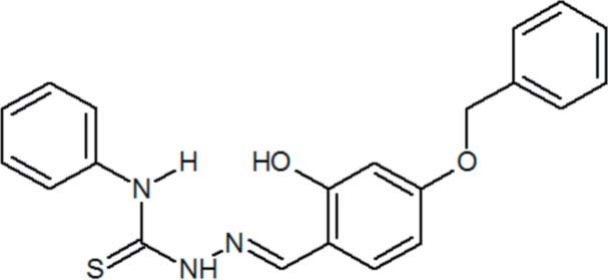


## Experimental

### 

#### Crystal data

C_21_H_19_N_3_O_2_S
*M* *_r_* = 377.45
Monoclinic, 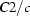

*a* = 24.099 (3) Å
*b* = 16.173 (2) Å
*c* = 9.8370 (11) Å
β = 95.906 (7)°
*V* = 3813.5 (8) Å^3^
*Z* = 8
Mo *K*α radiation
μ = 0.19 mm^−1^
*T* = 296 K
0.30 × 0.25 × 0.25 mm

#### Data collection

Bruker Kappa APEXII CCD diffractometer
Absorption correction: multi-scan (*SADABS*; Sheldrick, 2008 ▶) *T*_min_ = 0.945, *T*_max_ = 0.954
14236 measured reflections
3351 independent reflections
1848 reflections with *I* > 2σ(*I*)
*R*_int_ = 0.073

#### Refinement

*R*[*F* ^2^ > 2σ(*F* ^2^)] = 0.049
*wR*(*F* ^2^) = 0.148
*S* = 1.01
3351 reflections
256 parameters
2 restraints
H atoms treated by a mixture of independent and constrained refinement
Δρ_max_ = 0.19 e Å^−3^
Δρ_min_ = −0.22 e Å^−3^

### 

Data collection: *APEX2* (Bruker, 2004 ▶); cell refinement: *APEX2* and *SAINT* (Bruker, 2004 ▶); data reduction: *SAINT* and *XPREP* (Bruker, 2004 ▶); program(s) used to solve structure: *SIR92* (Altomare *et al.*, 1994 ▶); program(s) used to refine structure: *SHELXL97* (Sheldrick, 2008) ▶; molecular graphics: *ORTEP-3* (Farrugia, 1997 ▶); software used to prepare material for publication: *SHELXL97* and *publCIF* (Westrip, 2010 ▶).

## Supplementary Material

Crystal structure: contains datablock(s) global, I. DOI: 10.1107/S1600536811049658/jj2110sup1.cif

Structure factors: contains datablock(s) I. DOI: 10.1107/S1600536811049658/jj2110Isup2.hkl

Supplementary material file. DOI: 10.1107/S1600536811049658/jj2110Isup3.cml

Additional supplementary materials:  crystallographic information; 3D view; checkCIF report

## Figures and Tables

**Table 1:** Hydrogen-bond geometry (Å, °) *Cg*1 and *Cg*2 are the centroids of the C16–C21 and C1–C6 rings, respectively.

*D*—H⋯*A*	*D*—H	H⋯*A*	*D*⋯*A*	*D*—H⋯*A*
O2—H2*A*⋯N1	0.85 (4)	1.99 (4)	2.679 (3)	137 (4)
N2—H2*B*⋯S1^i^	0.85 (1)	2.55 (1)	3.392 (3)	170 (3)
C13—H13⋯O1^ii^	0.93	2.47	3.388 (4)	171
C5—H5⋯*Cg*^iii^	0.93	2.80	3.643 (4)	152
C12—H12⋯*Cg*^ii^	0.93	2.87	3.741 (4)	157

Symmetry codes: (i) 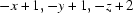; (ii) 
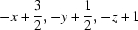
; (iii) 
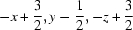
.

**Fig. 1.**
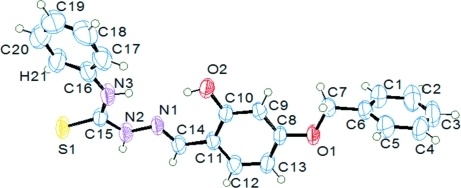
The molecular structure of the title compound with displacement ellipsoids are drawn at 50% probability level.

## supplementary crystallographic information

### Comment

Derivatives of hydrazinecarbothioamide are an important group of multidentate ligands with potential binding sites available for an extensive diversity of metal ions. A large number of studies have been devoted to the search for derivatives of hydrazinecarbothioamide, which have been used as drugs and have the ability to form complexes (Casas *et al.*, 2000). These compounds find substantial applications in different aspects of modern scientific research (Lukevics *et al.*, 1995).

The title compound (*E*)-2-[4-(benzyloxy)-2-hydroxybenzylidene]-*N*-\ phenylhydrazinecarbothioamide is found to exist in an *E* configuration having N3 and N1 atoms *cis* to each other with respect to C15–N2 bond (Fig. 1). The S1/C15/N12/N1 torsion-angle [-178.27 (19)°] suggests that the thionyl atom S1 is located *trans* to the azomethane nitrogen atom N1. The closeness of the C13═S1 bond distance [1.665 (3) Å] to the expected distance of a C═S bond (1.60 Å) (Allen *et al.*, 1987; Seena *et al.*, 2008) indicates that the compound exists in the thione form and it is further confirmed by the N1—N2 and N2—C15 bond distances. The hydrazinecarbothioamide moiety, (N1/N2/N3/C15/S1/C16), is nearly planar with a maximum deviation of 0.016 (3) and -0.016 (2) Å for atoms N3 and N2 from its least squares plane value. The three aromatic rings are twisted with a dihedral angle of 86.43 (17) Å between the least squares plane of the rings C1—C6 and C8—C13, 67.99 (19) Å between rings C1—C6 and C16—C21 and 29.77 (16) Å between rings C8—C13 and C16—C21, respectively.

An O2–H2A···N1 intramolecular hydrogen bond (Table 1), is observed which contributes to the planarity of the N1/C14/C11/C10/O2 group with a maximum deivation of 0.146 (2) Å for atom N1. Weak N—H···S, C—H···O and C—H···Cg π-ring intermolecular interactions (Table 1) are also observed.

### Experimental

The title compound was prepared by adapting a reported procedure (Joseph *et al.*, 2004) by refluxing a mixture of methanolic solutions of *N*-phenylhydrazinecarbothioamide (1.672 g, 10 mmol) and 4-(benzyloxy)-2-hydroxybenzaldehyde (2.282 g, 10 mmol) for four hours after adding 5 drops of acetic acid. Colorless crystals were collected, washed with few drops of methanol and dried over P_4_O_10_*in vacuo*. Single crystals of the title compound suitable for X-ray analysis were obtained by slow evaporation from its methanolic solution.

### Refinement

All H atoms on C were placed in calculated positions, guided by Fourier difference maps, with C—H bond distances 0.93Å (CH) or 0.97Å (CH_2_). H atoms were assigned as *U*_iso_=1.2Ueq. H3A and H2B hydrogen atoms were located from Fourier diffrence maps and restrained using the *DFIX* instruction. H2A was located from a Fourier difference map and freely refined.

### Crystal data

**Table:**

C_21_H_19_N_3_O_2_S	*F*(000) = 1584
*M**_r_* = 377.45	*D*_x_ = 1.315 Mg m^−^^3^
Monoclinic, *C*2/*c*	Mo *K*α radiation, λ = 0.71073 Å
Hall symbol: -C 2yc	Cell parameters from 1946 reflections
*a* = 24.099 (3) Å	θ = 5.0–28.5°
*b* = 16.173 (2) Å	µ = 0.19 mm^−^^1^
*c* = 9.8370 (11) Å	*T* = 296 K
β = 95.906 (7)°	Block, colorless
*V* = 3813.5 (8) Å^3^	0.30 × 0.25 × 0.25 mm
*Z* = 8	

### Data collection

**Table:**

Bruker Kappa APEXII CCD diffractometer	3351 independent reflections
Radiation source: fine-focus sealed tube	1848 reflections with *I* > 2σ(*I*)
graphite	*R*_int_ = 0.073
Detector resolution: 8.33 pixels mm^-1^	θ_max_ = 25.0°, θ_min_ = 2.5°
ω and φ scan	*h* = −28→28
Absorption correction: multi-scan (*SADABS*; Sheldrick, 2008)	*k* = −17→19
*T*_min_ = 0.945, *T*_max_ = 0.954	*l* = −11→11
14236 measured reflections	

### Refinement

**Table:**

Refinement on *F*^2^	Primary atom site location: structure-invariant direct methods
Least-squares matrix: full	Secondary atom site location: difference Fourier map
*R*[*F*^2^ > 2σ(*F*^2^)] = 0.049	Hydrogen site location: inferred from neighbouring sites
*wR*(*F*^2^) = 0.148	H atoms treated by a mixture of independent and constrained refinement
*S* = 1.01	*w* = 1/[σ^2^(*F*_o_^2^) + (0.0662*P*)^2^ + 0.0863*P*] where *P* = (*F*_o_^2^ + 2*F*_c_^2^)/3
3351 reflections	(Δ/σ)_max_ < 0.001
256 parameters	Δρ_max_ = 0.19 e Å^−^^3^
2 restraints	Δρ_min_ = −0.22 e Å^−^^3^

### Special details

**Table:**

Geometry. All e.s.d.'s (except the e.s.d. in the dihedral angle between two l.s. planes) are estimated using the full covariance matrix. The cell e.s.d.'s are taken into account individually in the estimation of e.s.d.'s in distances, angles and torsion angles; correlations between e.s.d.'s in cell parameters are only used when they are defined by crystal symmetry. An approximate (isotropic) treatment of cell e.s.d.'s is used for estimating e.s.d.'s involving l.s. planes.
Refinement. Refinement of *F*^2^ against ALL reflections. The weighted *R*-factor *wR* and goodness of fit *S* are based on *F*^2^, conventional *R*-factors *R* are based on *F*, with *F* set to zero for negative *F*^2^. The threshold expression of *F*^2^ > σ(*F*^2^) is used only for calculating *R*-factors(gt) *etc*. and is not relevant to the choice of reflections for refinement. *R*-factors based on *F*^2^ are statistically about twice as large as those based on *F*, and *R*- factors based on ALL data will be even larger.

### Fractional atomic coordinates and isotropic or equivalent isotropic displacement parameters (Å^2^)

**Table:**

	*x*	*y*	*z*	*U*_iso_*/*U*_eq_	
S1	0.49744 (4)	0.63473 (5)	1.03069 (9)	0.0709 (3)	
O1	0.76193 (9)	0.36630 (13)	0.4043 (2)	0.0729 (7)	
O2	0.65146 (10)	0.57793 (14)	0.5709 (2)	0.0737 (7)	
N1	0.59125 (9)	0.53647 (16)	0.7749 (2)	0.0511 (6)	
N2	0.55333 (10)	0.54839 (17)	0.8682 (2)	0.0550 (7)	
N3	0.57110 (11)	0.68512 (18)	0.8607 (3)	0.0664 (8)	
C1	0.79384 (15)	0.3466 (2)	0.1029 (4)	0.0845 (12)	
H1	0.7561	0.3571	0.0800	0.101*	
C2	0.82307 (18)	0.3021 (3)	0.0138 (4)	0.1015 (14)	
H2	0.8051	0.2830	−0.0683	0.122*	
C3	0.87770 (18)	0.2865 (3)	0.0463 (5)	0.0936 (13)	
H3	0.8974	0.2572	−0.0144	0.112*	
C4	0.90424 (15)	0.3129 (3)	0.1665 (5)	0.0871 (12)	
H4	0.9418	0.3010	0.1890	0.104*	
C5	0.87465 (14)	0.3584 (2)	0.2567 (4)	0.0787 (11)	
H5	0.8927	0.3771	0.3391	0.094*	
C6	0.81945 (14)	0.3754 (2)	0.2240 (3)	0.0630 (9)	
C7	0.78667 (13)	0.4237 (2)	0.3182 (3)	0.0681 (10)	
H7A	0.8110	0.4614	0.3730	0.082*	
H7B	0.7579	0.4558	0.2658	0.082*	
C8	0.72517 (12)	0.3956 (2)	0.4887 (3)	0.0562 (8)	
C9	0.70723 (11)	0.47536 (19)	0.4902 (3)	0.0530 (8)	
H9	0.7212	0.5143	0.4331	0.064*	
C10	0.66811 (11)	0.49850 (19)	0.5768 (3)	0.0496 (7)	
C11	0.64668 (11)	0.44162 (19)	0.6633 (3)	0.0493 (7)	
C12	0.66643 (13)	0.3615 (2)	0.6596 (3)	0.0701 (10)	
H12	0.6529	0.3225	0.7173	0.084*	
C13	0.70480 (14)	0.3371 (2)	0.5751 (3)	0.0751 (10)	
H13	0.7171	0.2827	0.5752	0.090*	
C14	0.60610 (11)	0.4627 (2)	0.7560 (3)	0.0534 (8)	
H14	0.5904	0.4205	0.8036	0.064*	
C15	0.54245 (11)	0.62420 (19)	0.9143 (3)	0.0513 (7)	
C16	0.57073 (14)	0.7713 (2)	0.8776 (3)	0.0637 (9)	
C17	0.61716 (16)	0.8131 (3)	0.8441 (3)	0.0847 (11)	
H17	0.6475	0.7836	0.8183	0.102*	
C18	0.6191 (2)	0.8979 (3)	0.8485 (4)	0.1049 (14)	
H18	0.6506	0.9255	0.8248	0.126*	
C19	0.5749 (2)	0.9422 (3)	0.8877 (4)	0.1129 (16)	
H19	0.5759	0.9996	0.8916	0.135*	
C20	0.5290 (2)	0.8994 (3)	0.9210 (4)	0.1051 (14)	
H20	0.4988	0.9287	0.9475	0.126*	
C21	0.52644 (15)	0.8151 (2)	0.9165 (3)	0.0814 (11)	
H21	0.4948	0.7878	0.9396	0.098*	
H2A	0.6261 (15)	0.589 (2)	0.621 (4)	0.110 (14)*	
H3A	0.5945 (10)	0.665 (2)	0.811 (3)	0.091 (12)*	
H2B	0.5385 (10)	0.5062 (10)	0.901 (3)	0.056 (9)*	

### Atomic displacement parameters (Å^2^)

**Table:**

	*U*^11^	*U*^22^	*U*^33^	*U*^12^	*U*^13^	*U*^23^
S1	0.0775 (6)	0.0587 (6)	0.0858 (6)	0.0045 (5)	0.0530 (5)	−0.0059 (4)
O1	0.0896 (15)	0.0552 (15)	0.0845 (14)	0.0196 (12)	0.0601 (12)	0.0102 (11)
O2	0.0914 (17)	0.0506 (16)	0.0884 (16)	0.0192 (13)	0.0544 (14)	0.0081 (12)
N1	0.0532 (14)	0.0533 (18)	0.0511 (14)	0.0046 (13)	0.0266 (11)	−0.0048 (11)
N2	0.0573 (15)	0.0492 (19)	0.0637 (16)	−0.0012 (14)	0.0309 (12)	−0.0037 (13)
N3	0.0770 (18)	0.0537 (19)	0.0762 (18)	−0.0036 (15)	0.0453 (15)	−0.0097 (14)
C1	0.083 (2)	0.090 (3)	0.086 (3)	0.020 (2)	0.035 (2)	−0.003 (2)
C2	0.102 (3)	0.119 (4)	0.089 (3)	0.022 (3)	0.040 (2)	−0.019 (2)
C3	0.097 (3)	0.091 (3)	0.103 (3)	0.013 (3)	0.063 (3)	−0.007 (3)
C4	0.068 (2)	0.084 (3)	0.118 (3)	0.017 (2)	0.051 (2)	0.011 (2)
C5	0.072 (2)	0.085 (3)	0.084 (2)	0.011 (2)	0.0340 (18)	0.0091 (19)
C6	0.075 (2)	0.052 (2)	0.069 (2)	0.0108 (18)	0.0398 (17)	0.0087 (17)
C7	0.077 (2)	0.063 (2)	0.072 (2)	0.0151 (18)	0.0440 (17)	0.0118 (16)
C8	0.0612 (18)	0.051 (2)	0.0612 (18)	0.0105 (16)	0.0308 (15)	−0.0032 (15)
C9	0.0587 (18)	0.052 (2)	0.0527 (17)	0.0045 (15)	0.0244 (14)	0.0025 (14)
C10	0.0536 (16)	0.044 (2)	0.0539 (17)	0.0046 (16)	0.0171 (13)	−0.0049 (14)
C11	0.0540 (17)	0.046 (2)	0.0511 (17)	−0.0008 (15)	0.0226 (13)	−0.0017 (13)
C12	0.088 (2)	0.052 (2)	0.079 (2)	0.0073 (19)	0.0501 (18)	0.0112 (16)
C13	0.094 (2)	0.047 (2)	0.094 (2)	0.0149 (19)	0.058 (2)	0.0077 (18)
C14	0.0564 (18)	0.054 (2)	0.0546 (17)	0.0014 (16)	0.0256 (14)	−0.0009 (15)
C15	0.0523 (17)	0.047 (2)	0.0569 (17)	0.0016 (16)	0.0181 (13)	−0.0051 (15)
C16	0.084 (2)	0.054 (2)	0.0575 (19)	−0.0069 (19)	0.0316 (16)	−0.0037 (16)
C17	0.107 (3)	0.069 (3)	0.086 (3)	−0.011 (2)	0.049 (2)	0.001 (2)
C18	0.146 (4)	0.075 (3)	0.101 (3)	−0.032 (3)	0.051 (3)	0.007 (2)
C19	0.180 (5)	0.052 (3)	0.115 (3)	−0.011 (3)	0.056 (3)	0.005 (2)
C20	0.147 (4)	0.058 (3)	0.118 (3)	0.005 (3)	0.047 (3)	−0.009 (2)
C21	0.099 (3)	0.055 (3)	0.097 (3)	0.000 (2)	0.043 (2)	−0.010 (2)

### Geometric parameters (Å, °)

**Table:**

S1—C15	1.665 (3)	C7—H7A	0.9700
O1—C8	1.360 (3)	C7—H7B	0.9700
O1—C7	1.428 (3)	C8—C9	1.360 (4)
O2—C10	1.345 (3)	C8—C13	1.395 (4)
O2—H2A	0.85 (4)	C9—C10	1.386 (3)
N1—C14	1.264 (4)	C9—H9	0.9300
N1—N2	1.374 (3)	C10—C11	1.388 (4)
N2—C15	1.342 (4)	C11—C12	1.382 (4)
N2—H2B	0.8501 (10)	C11—C14	1.445 (3)
N3—C15	1.341 (4)	C12—C13	1.363 (4)
N3—C16	1.403 (4)	C12—H12	0.9300
N3—H3A	0.8501 (11)	C13—H13	0.9300
C1—C6	1.366 (5)	C14—H14	0.9300
C1—C2	1.382 (4)	C16—C21	1.369 (4)
C1—H1	0.9300	C16—C17	1.376 (4)
C2—C3	1.346 (5)	C17—C18	1.373 (6)
C2—H2	0.9300	C17—H17	0.9300
C3—C4	1.354 (5)	C18—C19	1.372 (6)
C3—H3	0.9300	C18—H18	0.9300
C4—C5	1.403 (5)	C19—C20	1.371 (6)
C4—H4	0.9300	C19—H19	0.9300
C5—C6	1.364 (4)	C20—C21	1.364 (5)
C5—H5	0.9300	C20—H20	0.9300
C6—C7	1.498 (4)	C21—H21	0.9300
			
C8—O1—C7	118.2 (2)	C10—C9—H9	120.0
C10—O2—H2A	114 (3)	O2—C10—C9	116.8 (3)
C14—N1—N2	116.6 (2)	O2—C10—C11	122.1 (2)
C15—N2—N1	121.3 (2)	C9—C10—C11	121.2 (3)
C15—N2—H2B	119.9 (19)	C12—C11—C10	117.0 (2)
N1—N2—H2B	118.6 (19)	C12—C11—C14	119.7 (3)
C15—N3—C16	132.3 (2)	C10—C11—C14	123.3 (3)
C15—N3—H3A	110 (2)	C13—C12—C11	123.0 (3)
C16—N3—H3A	117 (2)	C13—C12—H12	118.5
C6—C1—C2	121.1 (4)	C11—C12—H12	118.5
C6—C1—H1	119.4	C12—C13—C8	118.6 (3)
C2—C1—H1	119.4	C12—C13—H13	120.7
C3—C2—C1	119.8 (4)	C8—C13—H13	120.7
C3—C2—H2	120.1	N1—C14—C11	122.4 (3)
C1—C2—H2	120.1	N1—C14—H14	118.8
C2—C3—C4	120.7 (3)	C11—C14—H14	118.8
C2—C3—H3	119.7	N3—C15—N2	114.3 (2)
C4—C3—H3	119.7	N3—C15—S1	126.4 (2)
C3—C4—C5	119.5 (4)	N2—C15—S1	119.3 (2)
C3—C4—H4	120.2	C21—C16—C17	119.3 (3)
C5—C4—H4	120.2	C21—C16—N3	124.2 (3)
C6—C5—C4	120.2 (4)	C17—C16—N3	116.4 (3)
C6—C5—H5	119.9	C18—C17—C16	120.6 (4)
C4—C5—H5	119.9	C18—C17—H17	119.7
C5—C6—C1	118.6 (3)	C16—C17—H17	119.7
C5—C6—C7	121.5 (3)	C19—C18—C17	120.3 (4)
C1—C6—C7	119.8 (3)	C19—C18—H18	119.8
O1—C7—C6	107.9 (3)	C17—C18—H18	119.8
O1—C7—H7A	110.1	C20—C19—C18	118.2 (4)
C6—C7—H7A	110.1	C20—C19—H19	120.9
O1—C7—H7B	110.1	C18—C19—H19	120.9
C6—C7—H7B	110.1	C21—C20—C19	122.1 (4)
H7A—C7—H7B	108.4	C21—C20—H20	118.9
O1—C8—C9	124.4 (3)	C19—C20—H20	118.9
O1—C8—C13	115.3 (3)	C20—C21—C16	119.5 (4)
C9—C8—C13	120.3 (2)	C20—C21—H21	120.3
C8—C9—C10	120.0 (3)	C16—C21—H21	120.3
C8—C9—H9	120.0		
			
C14—N1—N2—C15	167.7 (3)	C10—C11—C12—C13	−0.6 (5)
C6—C1—C2—C3	0.1 (6)	C14—C11—C12—C13	−179.8 (3)
C1—C2—C3—C4	1.0 (7)	C11—C12—C13—C8	0.1 (6)
C2—C3—C4—C5	−1.2 (6)	O1—C8—C13—C12	−178.0 (3)
C3—C4—C5—C6	0.4 (6)	C9—C8—C13—C12	0.5 (5)
C4—C5—C6—C1	0.6 (5)	N2—N1—C14—C11	−178.5 (2)
C4—C5—C6—C7	179.9 (3)	C12—C11—C14—N1	171.9 (3)
C2—C1—C6—C5	−0.9 (6)	C10—C11—C14—N1	−7.2 (5)
C2—C1—C6—C7	179.8 (3)	C16—N3—C15—N2	177.7 (3)
C8—O1—C7—C6	−173.0 (3)	C16—N3—C15—S1	−3.7 (5)
C5—C6—C7—O1	−91.8 (4)	N1—N2—C15—N3	0.5 (4)
C1—C6—C7—O1	87.5 (4)	N1—N2—C15—S1	−178.3 (2)
C7—O1—C8—C9	4.8 (5)	C15—N3—C16—C21	−24.8 (6)
C7—O1—C8—C13	−176.8 (3)	C15—N3—C16—C17	159.3 (3)
O1—C8—C9—C10	177.7 (3)	C21—C16—C17—C18	−0.5 (5)
C13—C8—C9—C10	−0.7 (5)	N3—C16—C17—C18	175.6 (3)
C8—C9—C10—O2	−178.7 (3)	C16—C17—C18—C19	0.6 (6)
C8—C9—C10—C11	0.2 (5)	C17—C18—C19—C20	−0.5 (7)
O2—C10—C11—C12	179.3 (3)	C18—C19—C20—C21	0.2 (7)
C9—C10—C11—C12	0.5 (4)	C19—C20—C21—C16	0.0 (6)
O2—C10—C11—C14	−1.5 (5)	C17—C16—C21—C20	0.2 (5)
C9—C10—C11—C14	179.6 (3)	N3—C16—C21—C20	−175.6 (3)

### Hydrogen-bond geometry (Å, °)

**Table:**

Cg1 and Cg2 are the centroids of the C16–C21 and C1–C6 rings, respectively.

**Table:**

*D*—H···*A*	*D*—H	H···*A*	*D*···*A*	*D*—H···*A*
O2—H2A···N1	0.85 (4)	1.99 (4)	2.679 (3)	137 (4)
N2—H2B···S1^i^	0.85 (1)	2.55 (1)	3.392 (3)	170 (3)
C13—H13···O1^ii^	0.93	2.47	3.388 (4)	171.
C5—H5···Cg^iii^	0.93	2.80	3.643 (4)	152
C12—H12···Cg^ii^	0.93	2.87	3.741 (4)	157

Symmetry codes: (i) −*x*+1, −*y*+1, −*z*+2; (ii) −*x*+3/2, −*y*+1/2, −*z*+1; (iii) −*x*+3/2, *y*−1/2, −*z*+3/2.
